# The Primary Care Practitioner’s Role in the Prevention and Management of Alcohol Problems

**Published:** 1994

**Authors:** Katherine A. Bradley

**Affiliations:** Katharine A. Bradley, M.D., M.P.H., is an acting instructor in the Department of Medicine, University of Washington, and a senior fellow in the Section of General Internal Medicine and Health Services Research and Development, Seattle Veterans Affairs Medical Center, Seattle, Washington

## Abstract

Medical practice in the United States has focused on diagnosing and treating alcohol abuse and dependence. A preventative approach to alcohol problems, however, requires that primary care practioners also identify patients whose patterns of alcohol consumption place them at risk for alcohol-related problems.

Primary care practitioners encounter a spectrum (figure 1) of alcohol-related problems. At one end are patients with severe alcohol dependence; at the other end are patients with hazardous patterns of alcohol consumption but no identifiable adverse consequences of drinking. In between are patients with a diverse array of alcohol-related problems.

Until recently, practitioners have focused largely on diagnosing alcohol disorders and referring patients with established alcohol abuse and dependence (for definitions of terms used in this article, see p. 99) to treatment specialists to prevent additional alcohol problems and minimize disability (Barnes et al. 1987). This type of intervention can be thought of as tertiary prevention (see the sidebar, p. 100). Recent research, however, has demonstrated that counseling patients with hazardous drinking patterns or mild alcohol-related problems that do not meet diagnostic criteria for alcohol disorders, or secondary prevention, also is effective in primary care settings (Bien et al. 1993). Although the efficacy of advising all patients about safe drinking practices, or primary prevention, is not known, it is practiced in some primary care settings (e.g., obstetrics).

**Terms Used in This Article**

**Alcohol abuse and alcohol dependence:** Refer to the disorders as defined by the criteria for substance abuse and dependence in the American Psychiatric Association’s 1994 *Diagnostic and Statistical Manual of Mental Disorders, Fourth Edition* (DSM–IV). These collectively can be referred to as “alcohol disorders.” According to DSM–IV, substance abuse criteria are as follows:

A maladaptive pattern of substance use leading to clinically significant impairment or distress, as manifested by one (or more) of the following, occurring within a 12-month period:

1. Recurrent substance use resulting in a failure to fulfill major role obligations at work, school, or home (e.g., repeated absences or poor work performance related to substance use; substance-related absences, suspensions, or expulsions from school; neglect of children or household)
2. Recurrent substance use in situations in which it is physically hazardous (e.g., driving an automobile or operating a machine when impaired by substance use)
3. Recurrent substance-related legal problems (e.g., arrests for substance-related disorderly conduct)
4. Continued substance use despite having persistent or recurrent social or interpersonal problems caused or exacerbated by the effects of the substance (e.g., arguments with spouse about consequences of intoxication, physical fights).

The symptoms have never met the criteria for substance dependence for this class of substance (pp. 182–183).

The criteria for substance dependence from DSM–IV are as follows:

A maladaptive pattern of substance use, leading to a clinically significant impairment or distress, as manifested by three (or more) of the following, occurring at any time in the same 12-month period:

1. Tolerance, as defined by either of the following:
  - a need for markedly increased amounts of the substance to achieve the intoxication or desired effect
  - markedly diminished effect with continued use of the same amount of the substance.
2. Withdrawal, as manifested by either of the following:
  - the characteristic withdrawal syndrome for the substance
  - the same (or a closely related) substance is taken to relieve or avoid withdrawal symptoms.
3. The substance is often taken in larger amounts or over a longer period than intended.
4. There is a persistent desire or unsuccessful efforts to cut down or control substance use.
5. A great deal of time is spent in activities necessary to obtain the substance (e.g., visiting multiple doctors or driving long distances), use the substance (e.g., chain-smoking), or recover from its effects.
6. Important social, occupational, or recreational activities are given up or reduced because of substance use.
7. The substance use is continued despite knowledge of having a persistent or recurrent physical or psychological problem that is likely to have been caused or exacerbated by the substance (e.g., continued drinking despite recognition that an ulcer was made worse by alcohol consumption) (p. 181).

**Alcohol problems and problem drinking:** Refer to the entire spectrum of alcohol-related problems, including those problems that do not meet DSM–IV criteria (Institute of Medicine 1990).

**Hazardous drinking:** Indicates patterns of alcohol consumption that place people at increased risk but have not yet resulted in problems (Saunders et. al. 1993*a*). Hazardous drinking may result from the amount of alcohol consumed daily, drinking to intoxication intermittently, and/or drinking in certain settings, such as before driving, that place people at risk.

**Three Levels of Prevention of Alcohol Problems in Primary Care Settings**

Primary care practitioners must fulfill several roles in managing the spectrum of alcohol-related problems. These roles can be thought of as three levels of prevention—primary, secondary, and tertiary (Institute of Medicine 1990), which is an approach used in the management of other chronic diseases. The goal of each level of prevention is the same: to prevent future adverse consequences that result from drinking. The target populations, however, differ. Tertiary prevention is directed at patients with established alcohol abuse or dependence. Secondary prevention is directed at patients with mild alcohol problems or hazardous drinking patterns that do not meet diagnostic criteria for alcohol abuse and dependence. Primary prevention targets all patients, regardless of their drinking habits.

**Tertiary Prevention**

Tertiary prevention of alcohol problems traditionally has been emphasized in U.S. medical education (Lewis et al. 1987). This level of prevention requires practitioners to identify patients with alcohol abuse and dependence and intervene by referring these patients to alcoholism treatment programs to prevent additional problems, such as trauma and cirrhosis.

**Secondary Prevention**

Secondary prevention of alcohol problems—brief interventions conducted by the primary care practitioner—is directed at preventing a progression to more severe problems, including dependence, and should be as important as tertiary prevention in primary care settings. Brief interventions are as effective as any other treatments of hazardous or mild problem drinking (Holder et al. 1991). Furthermore, because the number of hazardous drinkers in the population may exceed that of drinkers with severe alcohol dependence, the total societal cost (i.e., monetary and others) of the consequences resulting from hazardous drinking (including missed work and motor vehicle crashes) may exceed the cost of adverse consequences of all severely dependent drinkers (Kreitman 1986). Preventing consequences among hazardous drinkers with low-cost brief interventions, therefore, is a cost-effective approach to preventing alcohol problems.

**Primary Prevention**

Primary prevention of alcohol problems in primary care settings includes advising all patients about safe drinking practices, regardless of their drinking habits. There is no empirical evidence that primary prevention is effective (Moskowitz 1989). Nevertheless, primary prevention is standard practice in obstetrics where all pregnant women are advised to abstain from drinking alcohol (Bruce et al. 1993). Primary prevention of alcohol problems also may be rational in pediatric practices where anticipatory guidance is a standard component of primary care. —*Katharine A. Bradley*

ReferencesBruceFCAdamsMMShulmanHBAlcohol use before and during pregnancyAmerican Journal of Preventive Medicine926727319938257615HolderHLongabaughRMillerWRubonisAVThe cost effectiveness of treatment for alcoholism: A first approximationJournal of Studies on Alcohol526517401991166179910.15288/jsa.1991.52.517Institute of MedicineBroadening the Base of Treatment for Alcohol Problems: A Report of the Committee for the Study of Treatment and Rehabilitation for AlcoholismWashington, DCNational Academy Press1990KreitmanNAlcohol consumption and the preventive paradoxBritish Journal of Addiction813533631986346184610.1111/j.1360-0443.1986.tb00342.xLewisDCNivenRGCzechowiczDTrumbleJGA review of medical education in alcohol and other drug abuseJournal of the American Medical Association257212945294819873553640MoskowitzJMThe primary prevention of alcohol problems: A critical review of the research literatureJournal of Studies on Alcohol5054881989264807510.15288/jsa.1989.50.54

This article describes the roles of primary care practitioners in the identification and management of alcohol problems and suggests ways that practitioners can improve the prevention of alcohol problems in primary care settings.

## Epidemiology of Alcohol Disorders

### Alcohol Abuse and Dependence

These disorders are common in primary care settings. Their prevalence ranges from 11 to 20 percent in general medical clinics (Bradley 1992), 8 to 16 percent in family practice clinics (Leckman et al. 1984; Schorling et al. 1994), and 12 to 16 percent in obstetrics and gynecology practices (Halliday et al. 1986; Russell and Bigler 1979). Their prevalence in emergency departments is nearly 20 percent (Rund et al. 1981). Although few general pediatric patients meet the criteria for alcohol abuse or dependence (Duggan et al. 1991), an estimated one in eight children in the United States has a parent with a drinking problem (Kemper 1992).

Known risk factors for alcohol abuse and dependence include gender, age, family history, and ethnicity. Men are three to four times more likely than women to develop drinking problems (Bradley 1992). In men, heavy drinking often begins in the teenage years, with the risk of alcohol-related problems reaching a peak in the early twenties (Bradley 1992). Women tend to develop drinking problems at a slightly older age than men, but medical complications tend to occur at similar ages in men and women (Blume 1986). Youth who begin to use alcohol before age 16 (Smith 1993), or those with antisocial behavior (Vaillant 1983), are more likely to develop alcohol problems.

A genetic or cultural predisposition clearly places some people at higher risk than others (Schuckit 1985; Vaillant 1983). Men with two or more alcoholic relatives are about three times more likely to become alcohol dependent than those without (Vaillant 1983). Northern Europeans (Vaillant 1983) and some Native American groups (Institute of Medicine [IOM] 1990) also are at increased risk of developing drinking problems.

### Hazardous Drinking

Available evidence suggests that hazardous drinking is relatively common among primary care patients. Saunders and colleagues (1993*a*) reported that 19 percent of patients who drank alcohol consumed potentially hazardous amounts. Russell and Bigler (1979) found 19 percent of women in a gynecology practice to be heavy drinkers. Although the prevalence of hazardous drinking among pediatric primary care patients is not known, one-third of high school seniors engage in binge drinking at least every 2 weeks (Smith 1993).

Epidemiologic studies have identified several patterns of drinking that place people at increased risk for alcohol problems. Chronic heavy alcohol consumption can increase risk for cardiovascular disease, cirrhosis, and cancer (Bradley et al. 1993; National Institute on Alcohol Abuse and Alcoholism [NIAAA] 1993*a*). Consuming as few as two drinks daily has been associated with elevated blood pressure (Friedman 1990) and more than five drinks daily with increased mortality (Klatsky et al. 1992).

Episodic heavy drinking also is linked with adverse consequences, probably as a result of intoxication. Consuming more than four drinks per occasion has been associated with risk-taking behaviors (e.g., unplanned sexual activity and driving after drinking) and physical fights and blackouts in young adults (college students under 21 years old) (Wechsler and Isaac 1992).

Risks related to hazardous drinking often depend on the setting and the amount of alcohol consumed. Drinking when participating in water sports or before driving can be especially hazardous (Howland et al. 1990; Simel and Feussner 1990). For women, drinking more than 13 drinks a week at the time of conception is associated with abnormal fetal growth and development and fetal loss; more than 42 drinks a week significantly increases the risk of fetal alcohol syndrome (Bruce et al. 1993). For 14- to 16-year-olds, any drinking is associated with increased cigarette smoking and alcohol abuse later in life, and binge drinking is linked with property damage, poor academic performance, and violent behavior (Smith 1993). For parents, drinking is associated with accidental injury and child abuse (Macdonald and Blume 1986).

## Screening for Problem and Hazardous Drinking

Because many alcohol-dependent patients have no identifiable risk factors for alcohol problems, all primary care patients should be screened during the initial appointment and periodically thereafter. When the patient is a dependent child or is elderly, the practitioner should consider screening parents and caregivers as well (Macdonald and Blume 1986).

### Alcohol Abuse and Dependence

Identifying patients with alcohol abuse and dependence is complicated by the possibility that some patients will not recognize or admit that they have an alcohol problem. Consequently, several screening questionnaires have been developed to aid in identification (Bradley 1992), because they are a more sensitive means for detecting alcohol disorders than physical examination findings and laboratory tests (Skinner et al. 1986). For example, the CAGE (see Nilssen and Cone for this and other screening tests, pp. 136–139), which contains four questions, is probably the easiest for clinicians to remember (Ewing 1984).

### Hazardous Drinking and Mild Alcohol Problems

Although the CAGE is a good screening test for alcohol problems, it is an insensitive one for hazardous drinking (Wallace et al. 1987; Waterson and Murray-Lyon 1988). If the CAGE is used to identify hazardous drinking, it should be followed by questions about alcohol consumption or tolerance or both (Steinweg and Worth 1993). Although there is no consensus on a threshold for hazardous alcohol consumption, more than two drinks daily or more than three drinks per occasion can be considered potentially hazardous (Bradley et al. 1993).

Several newer screening questionnaires specifically designed to identify hazardous drinking as well as alcohol problems appear promising. The Alcohol Use Disorders Identification Test (AUDIT) is a 10-question test developed by the World Health Organization (Saunders et al. 1993*b*). The five-question TWEAK, originally developed to screen pregnant women, recently was found to be effective for other primary care patients (Chan et al. 1993). Although two versions of the TWEAK have been described, one appears to function better in clinical patients. Numerous instruments also have been developed specifically to identify hazardous and problem drinking by adolescents (NIAAA 1993*b*).

A laboratory test that measures the level of the liver enzyme gamma-glutamyl transferase (GGT) in the serum also will identify many patients with hazardous drinking. This GGT test, however, is generally less sensitive than screening questionnaires (Kristenson et al. 1983).

## Further Assessment of Patients Who Screen Positive

Adults who screen positive for problem or hazardous drinking require further assessment. In children, any drinking should trigger further assessment. An assessment interview can be done by a primary care nurse, social worker, or addictions specialist. Often, however, the practitioner will assess patients.

### Goals of the Assessment Interview

The primary goal of the assessment interview is to develop a clear understanding of the patient’s current drinking pattern and its associated risks. The interviewer should ascertain the patient’s typical and maximum daily alcohol consumption, any past history of heavy drinking, and whether the patient drinks before potentially dangerous activities (e.g., driving and boating). The interviewer also should identify coexisting medical and psychiatric problems complicated by alcohol consumption (e.g., hypertension and depression) and medications that interact with alcohol (e.g., the anticonvulsant phenytoin).

Another goal of the interview is to identify and evaluate the severity of alcohol-related problems. Although there is no generally accepted definition of problem drinking, the criteria for alcohol abuse and alcohol dependence in the *Diagnostic and Statistical Manual of Mental Disorders, Fourth Edition* (American Psychiatric Association 1994) (see this page) are a useful guide. The interviewer also should evaluate the severity of withdrawal symptoms, if present, and consider managing them medically.

In addition, the practitioner should use the interview to understand the patient’s perception of his or her drinking. Does the patient feel that he or she drinks too much? If so, why? Is the patient interested in drinking less? Has the patient ever tried to change his or her drinking habits? Future interventions can then reflect each patient’s readiness to change.

The final goal of the interview is to begin motivating problem drinkers to change their drinking habits (see brief interventions below).

### Suggested Approaches to the Assessment Interview

Several useful approaches have been described for assessing primary care patients with potential alcohol problems. One tactic is for the practitioner to begin with a discussion of the patient’s general lifestyle and stresses, followed by asking this question: “Where does your use of alcohol fit in?” (Rollnick et al. 1992). A similar approach is to ask the same question when discussing a specific health problem that a patient is having (Rollnick et al. 1992).

Patients who screen positive on a questionnaire such as the CAGE can be asked for clarification. The interviewer can say: “You indicated that you once felt you should cut down on your drinking. Can you tell me more about that?” (Bradley 1992).

Individual questions from screens such as the AUDIT or the TWEAK also may be helpful as part of an assessment interview.

Research on the validity of self-reported alcohol consumption suggests that the most valid information will be obtained when the interviewer and interviewee have good rapport and when the information obtained is confidential (Babor et al. 1991). In addition, defining what is meant by “a drink” (i.e., one glass of wine, one bottle of beer, or the equivalent of one shot of liquor); asking explicitly about each type of beverage consumed on a specific recent day; and reminding the patient to think of alcohol consumed between, as well as with, meals may increase the validity of self-reported alcohol consumption (NIAAA 1993*b*). Given its importance and multiple dimensions, the assessment interview may require several visits.

## Preventing Alcohol Problems

### Brief Interventions for Patients With Hazardous Drinking

If the assessment interview reveals that a patient drinks in a hazardous manner, the primary care practitioner, as well as nurses and other health care personnel, can use brief interventions to encourage a change in drinking habits (Bien et al. 1993). Offering a patient specific, non-judgmental feedback linking alcohol consumption and health can lead to change (Bien et al. 1993). Such feedback can include evidence of harm as a result of drinking (e.g., serum GGT indicating liver damage) (Kristenson et al. 1983) or information regarding potential harm (e.g., risk to the fetus from maternal drinking) (Bruce et al. 1993). Explicitly advising a patient to decrease consumption or abstain, in an empathetic and nonconfrontational manner, also can contribute to change (Bien et al. 1993; Walsh et al. 1992).

A practitioner’s optimism is another factor that can contribute to a patient’s motivation to change (Bien et al. 1993). Because motivation can waiver, patients deciding to change should be counseled to expect fluctuations in their resolve. All patients should be encouraged to follow up with the primary care practitioner whether or not they decrease their alcohol consumption.

## Managing Patients With Alcohol Problems

### Choosing a Goal

Although many experts in the United States believe that abstinence is the only acceptable goal for patients with alcohol problems, moderate drinking is an accepted treatment goal in other countries (IOM 1990).

#### Moderate Drinking in Patients With Problems

Patients with less severe dependence and those who believe they can drink moderately appear to be most successful at moderate drinking (Miller 1986; Rosenberg 1993). Studies of patients without severe dependence who were treated in programs specifically designed to teach them to drink in a controlled manner, report between 60 and 80 percent of these patients drinking without problems 1 to 2 years later (Miller 1986). Up to 5 to 20 percent of problem drinkers treated in abstinence-oriented programs have been reported to be drinking without problems at followup (Miller 1986).

#### When Is Moderate Drinking an Appropriate Goal?

Moderate drinking may be an acceptable goal for two groups of primary care patients with alcohol problems. First, patients with relatively mild alcohol problems, which do not meet criteria for alcohol dependence, may wish to try moderate drinking. Offering such patients a choice between abstinence and moderate drinking may motivate them to change their drinking (Miller 1985; Sanchez-Craig and Lei 1986).

Second, moderate drinking is an appropriate goal for primary care patients with alcohol dependence who recognize they drink too much but are unwilling to abstain. At a minimum, helping these patients lower their alcohol consumption might decrease alcohol-related morbidity.

Several studies of brief interventions aimed at moderate drinking have included significant numbers of patients with alcohol problems. Eighteen percent of patients studied by Wallace and colleagues (1987) screened positive on the CAGE or had a self-assessed drinking problem. Of patients with elevated GGT studied by Kristenson and colleagues (1981), 59 percent had tolerance, 30 percent had withdrawal symptoms, and 20 percent reported morning drinking. These studies demonstrated significant decreases in measures of morbidity, such as GGT, sick days, and hospital days, in men given moderate drinking interventions (Wallace et al. 1987; Kristenson et al. 1981).

Patients who try moderate drinking but are unable to control their consumption may subsequently become more accepting of abstinence as a treatment goal (Barnes et al. 1987). However, recurrent problem drinking is common among patients treated for alcohol abuse and dependence, regardless of whether the treatment goal is abstinence or moderate drinking (Rosenberg 1993).

### Referral

No *single* alcoholism treatment program has proved effective for all patients with alcohol problems, based on controlled clinical trials (IOM 1990). Nevertheless, most experts agree that all alcohol-dependent individuals should be referred to specialized alcoholism treatment programs or self-help groups (e.g., Alcoholics Anonymous or Rational Recovery).

Most treatment programs in this country are abstinence oriented, with a strong emphasis on group therapy. They vary from intensive, inpatient programs to weekly outpatient meetings. Studies are under way to match subgroups of patients to appropriate treatment programs (Project MATCH Research Group and NIAAA 1993); but, at present, the choice of treatment program often must be guided by availability, patient preferences, insurance coverage, and cost. Whenever possible, primary care practitioners should identify a colleague (e.g., alcoholism treatment specialist, social worker, or counselor) who is familiar with the entire range of local alcoholism treatment options to assist with referrals.

### Brief Interventions and Counseling To Increase Successful Referrals

Several interventions provided in the primary care setting can improve the completion rate of referrals for alcohol problems (Bien et al. 1993). Chafetz (1968) studied emergency department patients with alcohol dependence and found that more than 65 percent of patients who received brief empathetic counseling and assistance in obtaining social services followed through with referrals. In contrast, only 5 to 6 percent of patients who did not receive such attention followed through.

Once a patient is referred to a treatment program or self-help group, interventions such as letters, telephone calls, and followup appointments from primary care practitioners may help motivate patients to remain in treatment (Bien et al. 1993).

#### Counseling Patients Interested in Moderate Drinking

Although some self-help groups and alcoholism treatment programs concentrate on moderate drinking (e.g., Moderation Management and DrinkWise), most programs emphasize abstinence. Therefore, patients with alcohol problems who are interested in moderate drinking may not find a local treatment program that supports their goal. For these patients, primary care counseling also may be helpful.

To begin, the practitioner (or a clinic counselor) and the patient should identify a specific drinking goal (Bien et al. 1993). The practitioner also can make suggestions that will help the patient achieve the goal of moderate drinking (McIntosh and Sanchez-Craig 1984). A combination of self-monitoring (e.g., keeping a daily drinking diary) and other techniques that reduce alcoholic intake (e.g., interspersing nonalcoholic beverages, diluting drinks, and slowing the rate of drinking) can facilitate this goal (Alden 1988).

Some experts recommend 3 weeks of abstinence before moderate drinking is attempted. Such a period of abstinence might decrease tolerance and increase the patients’ confidence that they can control their drinking (Sanchez-Craig and Lei 1986).

#### Motivating Patients Not Interested in Change

If a problem drinker is not interested in changing the way he or she drinks and is not willing to accept referral, the practitioner should try to help motivate that patient to stop drinking in a harmful manner.

Limited research has been published on motivating primary care patients to change their drinking habits, but studies in treatment settings (Miller 1985), combined with evidence from brief intervention trials (Bien et al. 1993), suggest several ways to bring about change in primary care patients with alcohol abuse and dependence (Rollnick et al. 1992). Some patients may even decrease consumption in response to assessment, attempted referral, and followup alone (Elvy et al. 1988). Others will respond to the brief interventions directed at changing their drinking habits, described above.

For patients who recognize that they have a drinking problem but are not ready to change, practitioners may help stimulate change by exploring the patients’ ambivalence about drinking. Practitioners might ask such patients to describe what they like and dislike about drinking, thereby helping them articulate for themselves “their reasons for concern and the arguments for change” (Rollnick et al. 1992, p. 28).

### Patient Followup

Whichever approach is chosen for patients with hazardous drinking or alcohol problems—referral, brief intervention, or both—followup with the primary care practitioner likely will contribute to change (Bien et al. 1993).

For patients who are not ready to consider change at the time of the initial assessment, followup discussions after a period of self-assessment may help motivate patients to change. For patients who wish to change the way they drink, followup appointments allow the practitioner to monitor their progress and provide feedback. Reviewing changes in self-reported alcohol consumption, blood pressure, and laboratory tests such as the GGT may provide positive feedback and contribute to change (Bien et al. 1993). The practitioner can be an important source of motivation and support for patients when hazardous or problem drinking recurs. Primary care followup of patients with alcohol problems also can include prescription of medications to assist with abstinence or withdrawal (Chick et al. 1992; Hayashida et al. 1989).

## Improving Prevention of Alcohol Problems

As the gatekeeper in today’s health care environment, the primary care practitioner has a great responsibility for detecting and treating or referring patients with alcohol problems. To better recognize and evaluate patients who need help, practitioners must improve the care provided in the primary care setting.

### Prevention and Management of Alcohol Problems: Current Shortcomings

Despite the availability of useful screening tests for alcohol problems, patients with hazardous drinking patterns and alcohol problems often are not identified (Buchsbaum et al. 1992; Duggan et al. 1991; Leckman et al. 1984). Patients with inactive or mild alcohol problems are identified less often than patients with active problems or dependence. For example, internists are more likely to note the existence of alcohol problems in patients who have gastrointestinal complaints or have a previous diagnosis of alcohol abuse (Buchsbaum et al. 1992). In addition, women with alcohol problems are identified less often than their male counterparts (Buchsbaum et al. 1992).

Even when primary care patients with hazardous drinking patterns or alcohol problems are identified by screening, they may not receive appropriate care. Geller and colleagues (1989) reported that fewer than half of physicians in training felt a great responsibility to refer patients with alcohol problems; only 7 percent felt a great responsibility for followup with such patients. Although 93 percent of internists and family practitioners report counseling patients about alcohol use (Wells et al. 1984), this may overestimate alcohol counseling. Patient reports suggest that physicians frequently do not address alcohol use with their patients (Schorling et al. 1994). In addition, physicians appear most likely to counsel patients who have severe, active alcohol problems (Buchsbaum et al. 1993).

### Suggestions for Changes in Primary Care Practice

Certain methods for administering tests will improve the identification and management of patients with alcohol problems. Embedding screens in self-administered health questionnaires identifies a greater proportion of primary care patients with alcohol problems than if screening is left to physicians (Wallace et al. 1987). Response rates for such questionnaires appear to be better when the questionnaires are mailed to the patients, rather than administered by office receptionists (Kemper 1992; Wallace et al. 1987). Adolescents with alcohol abuse and dependence may be identified more often if interactive computer programs are used for screening (Paperny et al. 1990).

Programs in which nurses screen patients also may improve the identification, referral, and followup of patients with alcohol abuse and dependence. A study in which nurses screened general medical outpatients and referred them directly to an addictions counselor increased the rate of referral from 2 to 10 percent (Goldberg et al. 1991).

Similarly, when results from a diagnostic interview were provided to primary care physicians with explicit recommendations for counseling, the rate of counseling by physicians increased from 33 to 50 percent. Such screening and prompting especially increased physician counseling of patients least likely to be counseled: women and nondependent problem drinkers (Buchsbaum et al. 1993).

### Augmenting Education and Training

Educational programs should help primary care practitioners develop the attitudes and clinical skills needed to assess and manage patients with the entire spectrum of alcohol problems. At a minimum, more curricular time should be devoted to alcohol-related issues in medical schools, residency training programs, and continuing medical education courses (Adger et al. 1990).

Physicians in training have to develop a sense of responsibility, and optimism, toward their patients’ alcohol problems and confidence in their clinical skills related to caring for patients with these problems (Geller et al. 1989). Practitioners will acquire these qualities only if they have adequate clinical exposure to patients with alcohol problems (Bradley and Larson 1994).

In addition, practitioners likely will need experience-based learning, such as that learned from role-playing and supervised clinical practice, to develop the skills required for assessing and intervening with patients who drink too much (Adger et al. 1990; Warburg et al. 1987). Although knowledge per se does not appear to improve physician management of alcohol problems (Geller et al. 1989), secondary prevention and brief interventions have been relatively neglected in the U.S. medical literature (Bradley et al. in press). Therefore, practitioners may need increased didactic teaching about these topics.

### Focusing Future Research

Improving the prevention of alcohol problems in primary care settings also will require additional research. Several specific issues merit investigation.

Identifying the optimal screen for hazardous drinking and mild alcohol problems and evaluating which components of brief interventions are most effective in primary care settings are two significant issues. Studying how to distinguish patients who will respond to brief interventions by primary care practitioners from patients who will benefit from referral to treatment programs is another issue. Also, research must be conducted to determine whether primary prevention can reduce alcohol problems. Finally, medical educators must learn how best to prepare primary care practitioners for their diverse roles regarding the prevention of alcohol problems.

## Figures and Tables

**Figure 1:**
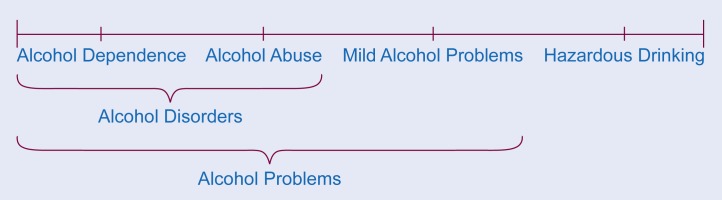
Spectrum of problems related to alcohol use.
